# Organocatalyzed chemoselective ring-opening polymerizations

**DOI:** 10.1038/s41598-018-22171-6

**Published:** 2018-02-27

**Authors:** Ning Zhu, Yihuan Liu, Junhua Liu, Jun Ling, Xin Hu, Weijun Huang, Weiyang Feng, Kai Guo

**Affiliations:** 1 College of Biotechnology and Pharmaceutical Engineering, Nanjing Tech University, Nanjing, 211800 China; 2 College of Materials Science and Engineering, Nanjing Tech University, Nanjing, 211800 China; 3 State Key Laboratory of Materials-Oriented Chemical Engineering, Nanjing Tech University, Nanjing, 211800 China; 4 Jiangsu National Synergetic Innovation Center for Advanced Materials, Nanjing Tech University, Nanjing, 211800 China; 5 Department of Polymer Science and Engineering, Key Laboratory of Macromolecular Synthesis and Functionalization of the Ministry of Education, Zhejiang University, Hangzhou, 310027 China; 6 Zhejiang Center for Drug & Cosmetic Evaluation, Hangzhou, 310012 China

## Abstract

A novel metal-free and protecting-group-free synthesis method to prepare telechelic thiol-functionalized polyesters is developed by employing organocatalysis. A scope of Brønsted acids, including trifluoromethanesulfonic acid (**1**), HCl.Et_2_O (**2**), diphenyl phosphate (**3**), γ-resorcylic acid (**4**) and methanesulfonic acid (**5**), are evaluated to promote ring-opening polymerization of ε-caprolactone with unprotected 6-mercapto-1-hexanol as the multifunctional initiator. Among them, diphenyl phosphate (**3**) exhibits great chemoselectivity and efficiency, which allows for simply synthesis of thiol-terminated poly(ε-caprolactone) with near-quantitative thiol fidelity, full monomer conversion, controlled molecular weight and narrow polydispersity. Kinetic study confirms living/controlled nature of the organocatalyzed chemoselective polymerizations. Density functional theory calculation illustrates that the chemoselectivity of diphenyl phosphate (**3**) is attributed to the stronger bifunctional activation of monomer and initiator/chain-end as well as the lower energy in hydroxyl pathway than thiol one. Moreover, series of tailor-made telechelic thiol-terminated poly(δ-valerolactone) and block copolymers are efficiently generated under mild conditions.

## Introduction

Organocatalysis has been deeply investigated and widely applied in the chemical transformations^1^. Numerous excellent contributions were reported in this blooming research area^2–8^. In polymer chemistry, organocatalysis provided remarkable opportunities in precision well-defined polymers^9–11^. The features of the use of small organic molecules as the catalyst or initiator in ring-opening polymerization (ROP) of cyclic monomers were explored by many groups^12–16^. The classes of organocatalysts have been continuously developed based on the general polymerization mechanisms of electrophilic monomer activation, nucleophilic monomer activation, initiator or chain-end activation and bifunctional activation of monomer and initiator/chain end^17,18^. Despite tremendous progress was made, bottlenecks still remained in organocatalyzed ROP, such as chemoselectivity, stereoselectivity and switchable catalysis^18,19^.

Chemoselective polymerization in the presence of multifunctional initiator/monomer is the ideal yet challenging green synthetic strategy to prepare functional polymers^20,21^. Thiol-functionalized polymers have significant applications in polymer chemistry and nanoscience, which requires quantitative thiol fidelity, controlled molecular weight and narrow polydispersity^22–25^. However, due to its special chemical activities and incompatibilities with many polymerization processes, protected thiol strategies in macromolecular design and synthesis were established to prevent the unwanted side reactions^26^. Traditionally, tedious protecting/deprotecting steps were incorporated during ring-opening polymerization with mercapto alcohol as multifunctional initiator^27–29^. Thus, quantitatively chemoselective and highly efficient synthetic strategies are extremely desirable to meet the requirement of green and sustainable chemistry.

**Figure 1:**
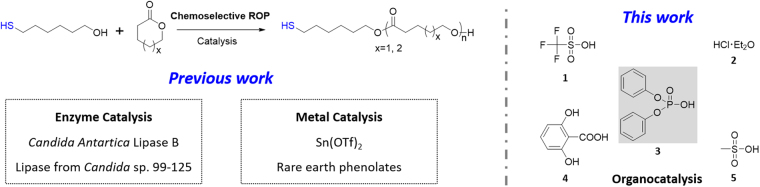
Chemoselective ring-opening polymerizations in previous and this work.

Since the initial discovery in 2005, protecting-group-free ring-opening polymerization method has been presented by using enzyme or metal catalysis to directly obtain thiol-functionalized polyester (Fig. 1)^30–35^. However, it has been suffering scientific and engineering problems, including but not limited to low thiol fidelity, long-time consuming process, poor control of molecular weight and polydispersity. Novel catalysis should be developed to satisfy the supreme demand of chemoselective polymerizations.

Organic acids are an “old” but simple and effective organocatalyst family for ring-opening polymerization^36^. The fundamental activated monomer mechanism (protonation of the cyclic monomer and subsequent ring-opening by a nucleophile) makes organic acid chemoselective catalyst candidate toward hydroxyl and thiol. Here, we evaluate the chemoselectivities of several commercial available Brønsted acids for mercapto alcohol initiated ring-opening polymerization, including trifluoromethanesulfonic acid (**1**), HCl.Et_2_O (**2**), diphenyl phosphate (**3**), γ-resorcylic acid (**4**) and methanesulfonic acid (**5**) (Scheme 1). Among them, diphenyl phosphate (**3**), which was developed by Kakuchi^37–41^, Bourissou^42^ and co-workers, is found to be the relative higher chemoselective catalyst. Density functional theory (DFT) calculation reveals a stronger bifunctional activation of monomer and initiator/chain-end and lower energy in hydroxyl pathway than thiol route. By employing this metal-free and protecting-group-free green synthetic protocol, tailor-made telechelic thiol-terminated poly(ε-caprolactone) (PCLSH), poly(δ-valerolactone) (PVLSH) and block copolylactones (PCL-*b*-PVLSH and PVL-*b*-PCLSH) are simply prepared, with near-quantitative thiol fidelity, full monomer conversion, controlled molecular weight and narrow polydispersity. Moreover, the resultant thiol-functionalized polymers shows interesting application in stabilizing metal nanoparticles.

## Results and Discussion

The primary requirements for broad applications of thiol-functionalized polymers are quantitative thiol fidelity, controlled molecular weight and narrow polydispersity, which have not yet been achieved by the previous methods^30–35^. To address these challenges, a scope of Brønsted acids, including trifluoromethanesulfonic acid (**1**), HCl.Et_2_O (**2**), diphenyl phosphate (**3**), γ-resorcylic acid (**4**) and methanesulfonic acid (**5**), were investigated respectively in ε-caprolactone (CL) polymerizations initiated by 6-mercapto-1-hexanol (MH) as the multifunctional initiator. The polymerization results were summarized in Table 1. Under the initial conditions ([CL]:[MH]:[Catalyst] = 50:1:0.5, [CL] = 2 mol/L), all acids enabled full monomer conversions for different reaction temperatures and times. The fractions of desirable thiol-terminated polymer in the product, defined as thiol fidelity, were ranged between 69% and 96%. The molecular weights (*M*_n,NMR_) according to NMR analysis agreed with the theoretical values (*M*_n,theo_) and the molecular weight distributions (*Ð*_M_) were very narrow (<1.10). It was noteworthy that no large distinction was observed between the strong acid and weak acid (**1** vs **5**, **2** vs **4**) with respect to the reaction temperature, time, thiol fidelity, molecular weight and polydispersity. As the acidity decreased from **1** to **5**, moderate acid of **3** exhibited relative higher chemoselectivity (96% thiol fidelity) (Table 1, run 4). It might be correlated with the structure of diphenyl phosphate (**3**).

**Table 1:** Results of Brønsted acids catalyzed chemoselective ROP of CL.

Run	Cat	[CL]:[MH]:[Cat]	Temp. °C	Time min	Conv. %	Thiol fidelity^b^ %	*M*_n,theo_^c^ g/mol	*M*_n,NMR_^d^ g/mol	*Ð*_M_^e^
1	**1**	50:1:0.5	25	90	99	80	5840	5160	1.03
2^a^	**2**	50:1:0.5	0	1080	93	69	5440	3440	1.03
3	**3**	30:1:0.5	50	70	95	96	3380	3780	1.04
4	**3**	50:1:0.5	50	150	95	96	5550	5400	1.09
5	3	80:1:0.5	50	360	98	91	9070	8460	1.08
6	**3**	100:1:0.5	80	180	95	91	10970	9610	1.10
7^f^	**4**	50:1:1	25	1920	95	70	5550	4590	1.07
8	**5**	50:1:0.5	25	120	93	72	5440	4360	1.03

^a^Solvent was dichloromethane; ^b^thiol fidelity was calculated by integral comparison (H^w^/H^a^) in ^1^H NMR; ^c^*M*_n,theo_ was calculated by combination of [CL]:[MH] feed ratio, conversion and molecular weight of initiator and monomer; ^d^*M*_n,NMR_ was calculated by combination integral comparison (H^r+g^/H^a^) in ^1^H NMR, molecular weight of initiator and monomer; ^e^*Ð*_M_ was obtained by SEC; ^f^[CL] = 3.0 mol/L.

**Figure 2:**
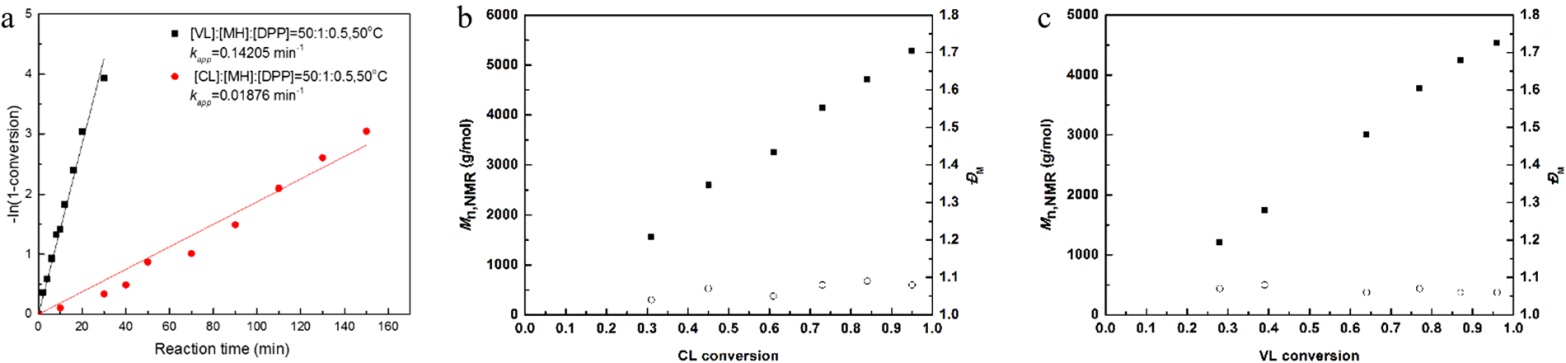
Semilogarithmic kinetic plots for diphenyl phosphate (**3**) catalyzed chemoselective ROP of CL and VL (**a**); dependence of *M*_*n,NMR*_ and *Ð*_*M*_ on the CL (**b**) and VL conversion (**c**).

Subsequently, diphenyl phosphate (**3**) was chosen as the model investigation organic acid. The kinetics studies elucidated the linear increases between –ln(1-conversion) and reaction time, which indicated polymerization rate to be first order in monomer concentration (Fig. 2a). Linear dependences of molecular weight (*M*_n,NMR_) and monomer conversion were plotted in Fig. 2b, while the molecular weight distributions (*Ð*_M_) kept narrow. To further examine the versatility of diphenyl phosphate (**3**), we performed experiments with different [CL]:[MH] feed ratio to produce thiol-terminated poly(ε-caprolactone) (PCLSH) with varied molecular weights. *M*_n,NMR_ increased as the elevating monomer feed ratio from 3000 to 10000 g/mol with narrow molecular weight distributions (*Ð*_M_ < 1.10) (Table 1, run 3–6). All thiol fidelities were near-quantitative, which cannot be done by the previous enzyme or metal catalysis^30–35^.

**Figure 3:**
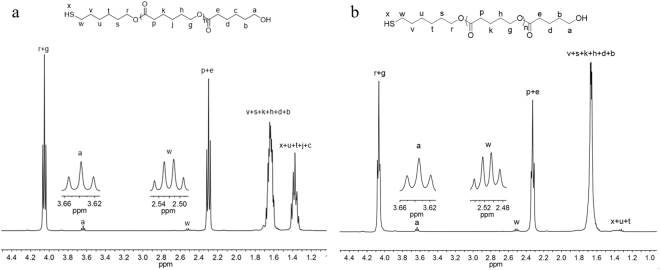
^1^H NMR of PCLSH (Table 1, run 4) (**a**) and PVLSH (Table 2, run 10) (**b**).

**Figure 4:**
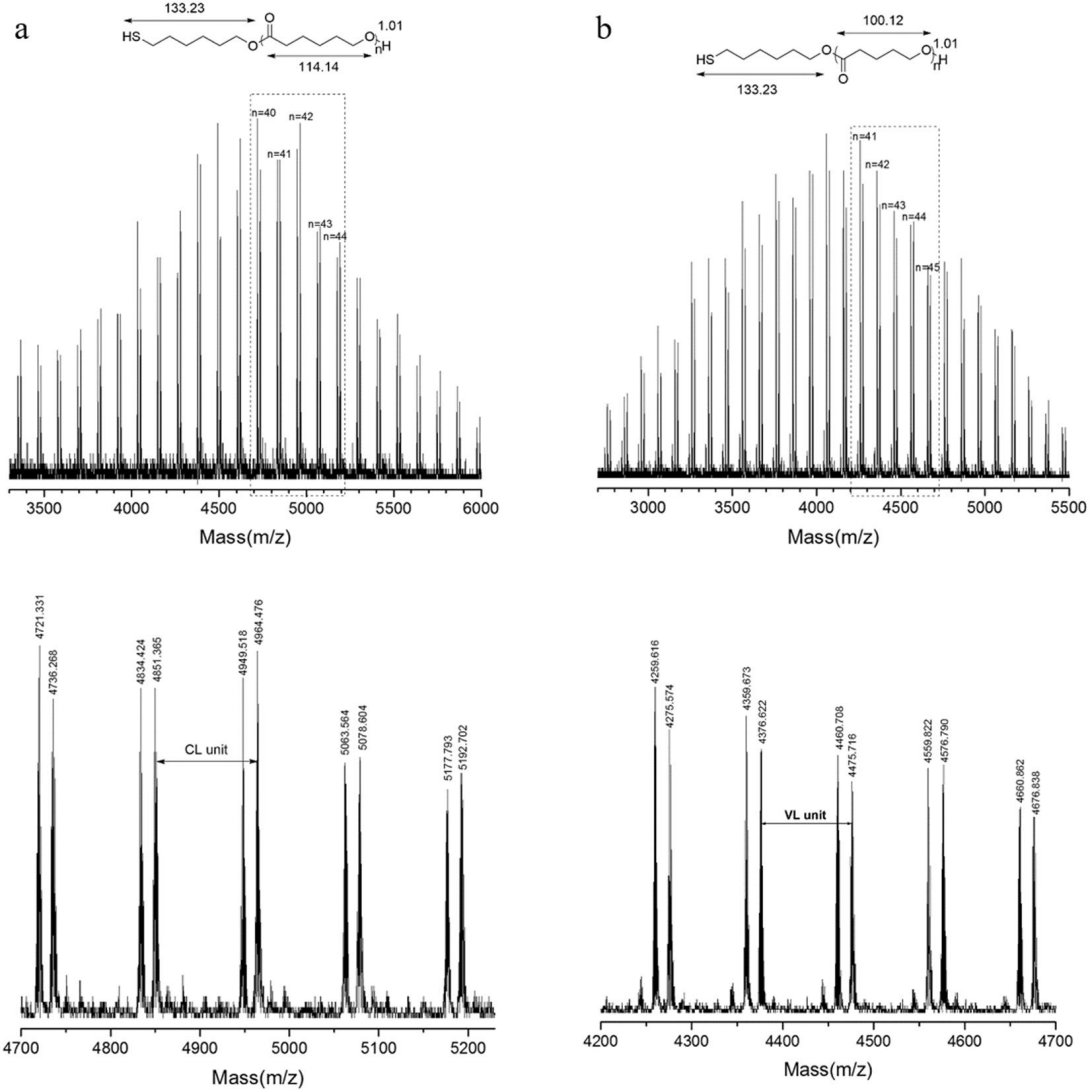
MALDI TOF MS of PCLSH (Table 1, run 4) (**a**) and PVLSH (Table 2, run 10) (**b**).

**Figure 5:**
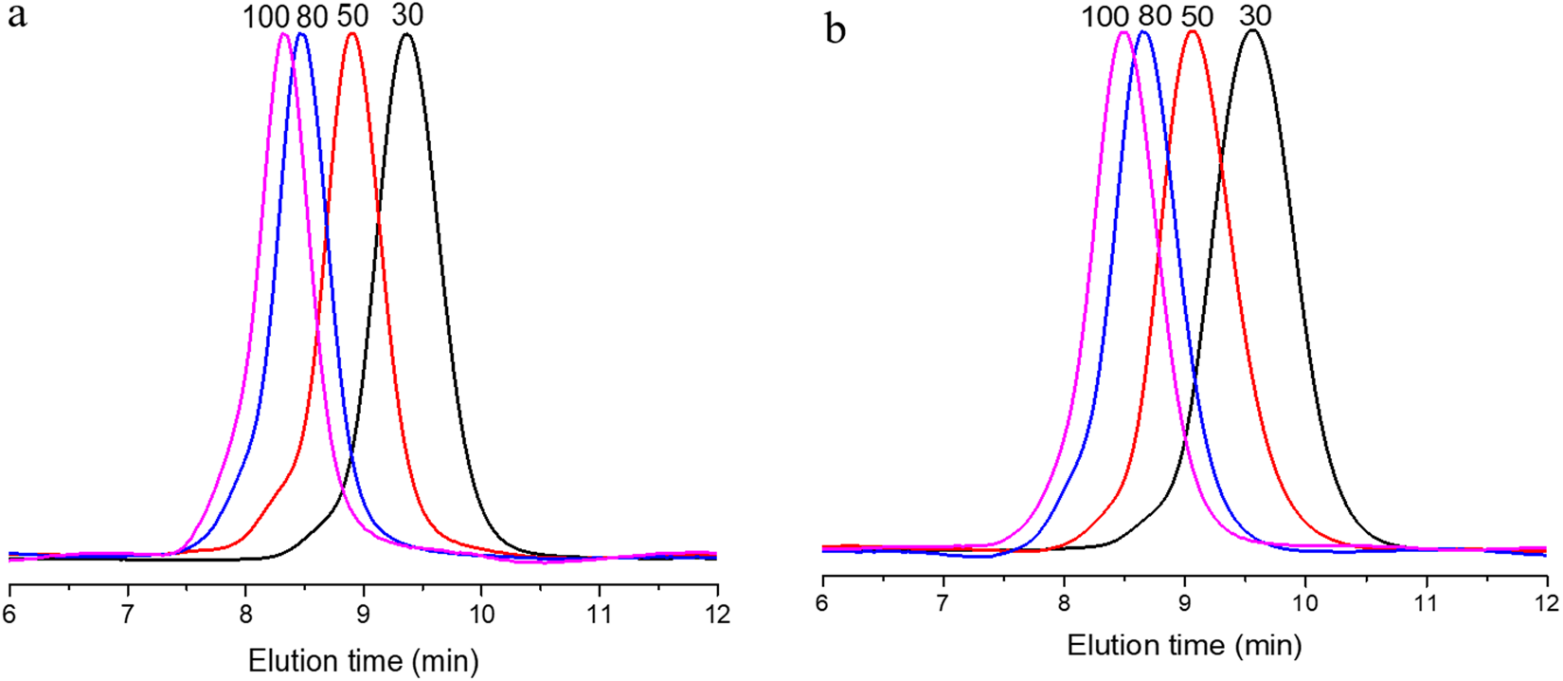
SEC of PCLSH (Table 1, run 3–6) (**a**) and PVLSH (Table 2, run 9–12) (**b**).

The chemical structures of PCLSH were characterized by NMR, MALDI TOF MS and SEC. In Fig. 3a, besides the feature proton signals in PCL backbone, the appearance of quartet peak at around 2.5 ppm (H^w^) revealed the presence of thiol as polymer end group, which was assigned to the methylene proton signals adjacent to the thiol. The other end group of hydroxyl could be validated by the triplet peak at 3.6 ppm (H^a^). Thiol fidelity was obtained to be 96% for PCL (Table 1, run 4) by the integral comparison between H^w^ and H^a^. *M*_n,NMR_ were calculated to be 5400 g/mol, which agreed with the theoretical values. The proton signals of thiol (H^x^) and others in initiator were overlapped by those of polymer backbones. The direct evidence was supplied by ^1^H-^1^H COSY (Figure S1a). The coupling signals of area B and C confirmed the presence of H^x^ and H^v^. ^13^C NMR (Figure S2a) showed that all signals were fully assigned and no thiolester and disulfide structure existed. MALDI TOF MS provided detailed polymer information of molecular weight. As depicted in Fig. 4a, two series of main peaks cationized by Na^+^ and K^+^ were clearly observed with separation of 114 (CL unit). The molecular weights were consistent with the theoretical values of PCLSH. Signals corresponding to the disulfide structure were not detected. The molecular weight distributions were measured by SEC. The symmetrical monomodal SEC traces of PCLSH elucidated their narrow polydispersities (Fig. 5a).

**Figure 6:**
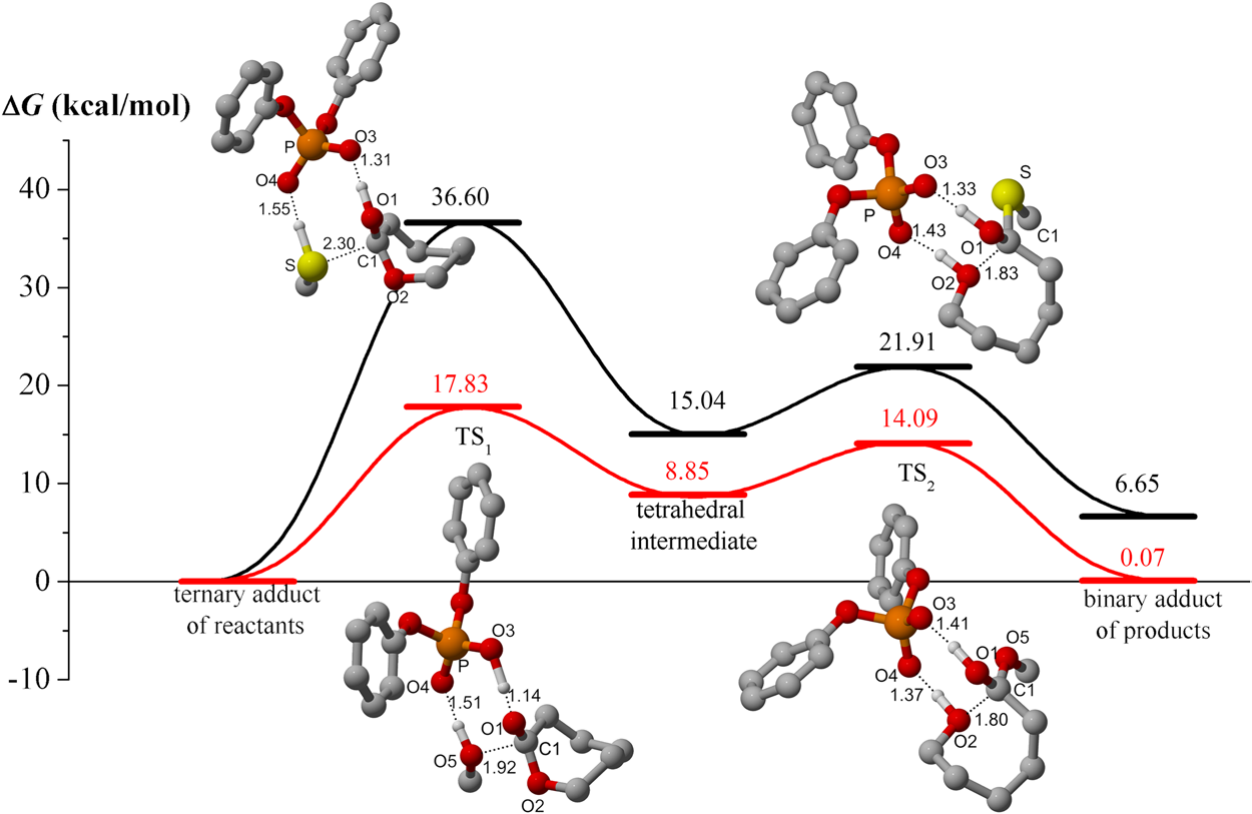
Calculated Gibbs free energy profiles of the organocatalyzed ring opening reactions initiated by methanol (red line) and methanthiol (black line) respectively. Optimized geometries were illustrated by 3D models where some hydrogen atoms are neglected for clarity.

Our next concern was the chemoselective polymerization mechanism. According to the reports of of Penczek^36^, Kakuchi^41^, and Bourissou^42^, Brønsted acids catalyzed CL polymerization initiated by unprotected MH was assumed to obey activated monomer mechanism. In the last decade, ring-opening polymerization process has been explored by using computational studies^43–49^. To get better understanding of the great chemoselectivity of diphenyl phosphate (**3**), we carried out DFT calculations to compare two model reactions of CL ring-opening with methanol (CH_3_-OH) or methanthiol (CH_3_-SH) (Fig. 6 and details in Figure S3). Cooperative bifunctional activation of initiator and monomer was involved in transition state TS_1_ for the nucleophilic addition step. It was clearly seen that OH in methanol was closer to carbonyl of CL than SH (O_5_-C_1_ 1.92 Å vs S-C_1_ 2.30 Å). The distance of hydrogen bond between P=O and OH was shorter than that between P=O and SH (O_4_-H 1.51 Å vs O_4_-H 1.55 Å), which indicated stronger initiator/chain-end activation by diphenyl phosphate (**3**) in the presence of methanol as the initiator. Energy of TS_1_ (OH pathway) was lower by about 18 kcal/mol than that of SH pathway. Ring-opening of CL proceeded via transition state TS_2_. Accompanied with proton transfer from acid to the endocyclic oxygen, endocyclic C–O bond was cleaved. Lower energy of TS_2_ in OH pathway was obtained in comparison with SH (14.09 Kcal/mol vs 21.91 kcal/mol). Therefore, it was proposed that the great chemoselectivity of diphenyl phosphate (**3**) was resulted from the stronger bifunctional activation of monomer and initiator/chain-end and lower energy in OH pathway than SH route.

**Figure 7:**
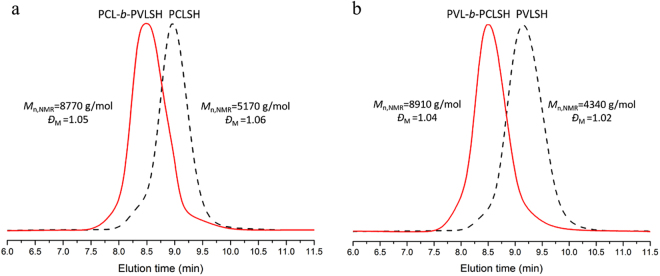
SEC traces of PCL-*b*-PVLSH (**a**) and PVL-*b*-PCLSH (**b**).

**Figure 8:**
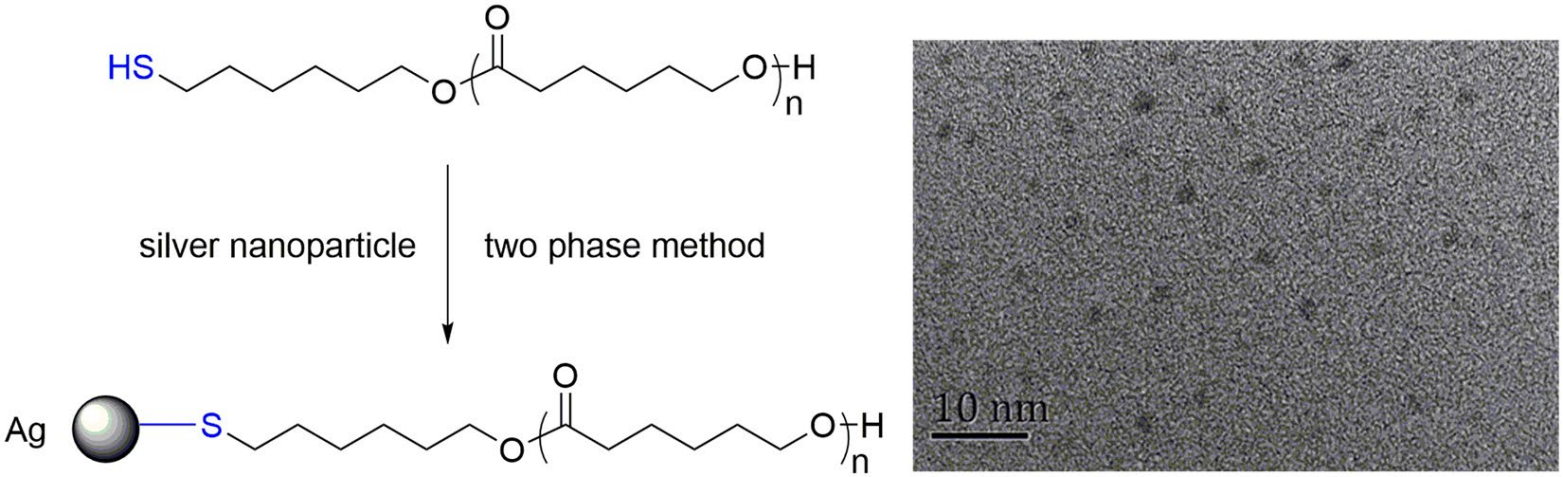
TEM of thiol-terminated PCL stabilized silver nanoparticles via two phase method.

Then, our attention was paid on the application of this metal-free and protecting-group-free green synthetic approach. By using diphenyl phosphate (**3**) as the organic acid catalyst, the monomer was extended into δ-valerolactone (VL). Under similar reaction conditions, well-defined thiol-terminated poly(δ-valerolactone) (PVLSH) were prepared with quantitative thiol fidelity, broad molecular weight range and narrow polydispersities (Table 2). The linear increases between –ln(1-conversion) and reaction time were recorded in Fig. 2a. The apparent polymerization rate constant of VL (*K*_app_ = 0.14205 min^−1^) was larger than that of CL (*K*_app_ = 0.01876 min^−1^), which was consistent with the previous reports^41,42^.The molecular weight (*M*_n,NMR_) increased linearly with the monomer conversion (Fig. 2c). The chemical structure of PVLSH was demonstrated by ^1^H NMR (Fig. 3b),^1^H-^1^H COSY (Figure S1b),^13^C NMR (Figure S2b), MALDI TOF MS (Fig. 4b) and SEC (Fig. 5b). To further confirm the living/controlled nature of diphenyl phosphate (**3**) catalyzed chemoselective polymerization, we carried out the chain extensions in one pot. PCLSH (*M*_n,NMR_ = 5170 g/mol, *Ð*_M_ = 1.06) was first synthesized from polymerization ([CL]:[MH]:[Catalyst] = 50:1:0.5, [M] = 2 mol/L) for 150 min at 50 °C. A further polymerization was conducted by addition of 50 equivalent of VL. After another 30 min, PCL-*b*-PVLSH (*M*_n,NMR_ = 8770 g/mol, *Ð*_M_ = 1.05) was obtained with thiol fidelity of 99%. By alternating the monomer induction sequence, PVL-*b*-PCLSH was also synthesized (*M*_n,NMR_ = 8910 g/mol, *Ð*_M_ = 1.04, 99% thiol fidelity). The shifts of SEC traces toward higher molecular weight region indicated the formation of block structures (Fig. 7) ^1^H NMR **(**Figure S4) and ^13^C NMR (Figure S5) illustrated the chemical structures of block copolymers. The polyesters with thiol functionality enabled multiple promising applications^22–25^. The resultant PCLSH (*M*_n,NMR_ = 5400 g/mol, *Ð*_M_ = 1.09, thiol fidelity = 96%) protected silver nanoparticles were prepared through two phase method^25^. Well-dispersed silver nanoparticles were clearly shown in TEM (Fig. 8), which was promising in biospecific labeling.

**Table 2:** Results of diphenyl phosphate (**3**) catalyzed chemoselective ROP of VL.

Run^a^	Cat	[VL]:[MH]:[Cat]	Temp. °C	Time min	Conv. %	Thiol fidelity^b^ %	*M*_n,theo_^c^ g/mol	*M*_n,NMR_^d^ g/mol	*Ð*_M_^e^
9	**3**	30:1:0.5	50	10	95	99	2980	3120	1.03
10	**3**	50:1:0.5	50	30	99	99	5080	4740	1.04
11	**3**	80:1:0.5	50	90	97	99	7890	6240	1.14
12	**3**	100:1:0.5	50	150	96	99	9730	8780	1.14

^a^All polymerizations were conducted in toluene ([VL] = 2.0 mol/L); ^b^thiol fidelity was calculated by integral comparison (H^w^/H^a^) in ^1^H NMR; ^c^*M*_n,theo_ was calculated by combination of [VL]:[MH] feed ratio, conversion and molecular weight of initiator and monomer; ^d^*M*_n,NMR_ was calculated by combination integral comparison (H^r+g^/H^a^) in ^1^H NMR, molecular weight of initiator and monomer; ^e^*Ð*_M_ was obtained by SEC.

## Conclusions

A novel metal-free and protecting-group-free green synthetic approach to thiol-functionalized polymers was developed by the utility of organocatalysis. Trifluoromethanesulfonic acid (**1**), HCl.Et_2_O (**2**), diphenyl phosphate (**3**), γ-resorcylic acid (**4**) and methanesulfonic acid (**5**) all showed chemoselective activity toward hydroxyl and thiol. Diphenyl phosphate (**3**) achieved relative higher quantitative chemoselectivity in synthesis of well-defined thiol-terminated *homo*- and *block*- polyesters. Density functional theory calculations explained that it was attributed to stronger bifunctional activation of monomer and initiator/chain-end and lower energy in hydroxyl pathway than thiol. This simple and green synthesis method would meet the supreme demand for the mercapto-polymers synthesis and applications. We believe that this work would get deep understanding of organocatalysis and chemoselective polymerization.

## Methods

### Materials

ε-Caprolactone (CL) (J&K, 99%) and δ-valerolactone (VL) (J&K, 99%) was distilled over CaH_2_ under reduced pressure. Toluene (Sinopharm chemical Reagent, 99.5%) was refluxed over sodium under an argon atmosphere. Trifluoromethanesulfonic acid (**1**) (J&K, 99%), HCl.Et_2_O (**2**) (prepared according to literature)^50^, diphenyl phosphate (**3**) (TCI, 99%), γ-resorcylic acid (**4**) (J&K, 99%), methanesulfonic acid (**5**) (J&K, 99%) and 6-Mercapto-1-hexanol (MH) (TCI, 98%) were stored under argon atmosphere. Lithium hydroxide (LiOH) (J&K, 99%), benzyl glycidyl ether (TCI, 97%), silver nitrate (AgNO_3_) (MACKLIN, 99.8%), sodium borohydride (NaBH_4_) (Aladdin, 98%), tetraoctylammonium bromide ((n-C_8_H_17_)_4_NBr) (MERYER, 98%) and other chemicals were purchased and used without purification.

### Synthetic Procedures

#### Chemoselective ring-opening polymerization

Polymerizations were performed by using Schlenk technique. Take CL polymerization for example. MH (0.0403 g, 0.30 mmol), DPP (0.0375 g, 0.15 mmol) and 5.8 mL toluene were transferred into the previously flamed and argon-purged ampoule. The reaction proceeded at 50 °C by addition of CL (1.7121 g, 15.0 mmol). Aliquots were taken and quenched by triethylamine for conversion detection by ^1^H NMR. The polymerization was ended by adding cold methanol with triethylamine. The product was precipitated, filtrated and dried in vacuum at room temperature.

#### Metal nanoparticle preparation

The silver nanoparticles were prepared by two-phase method. 5 mL (0.10 mol/L) (n-C_8_H_17_)_4_NBr in toluene and 5 mL (0.05 mol/L) aqueous solution of AgNO_3_ was mixed under rapid stirring. Thiol-terminated PCL (*M*_n,NMR_ = 5400, *Ð*_M_ = 1.09) (0.2650 g, 0.05 mmol) in 5 mL toluene was added followed by slow addition of 5.0 mL freshly prepared aqueous solution of NaBH_4_ (0.25 mol/L). The organic phase of reaction mixture was separated and concentrated by evaporation at room temperature and finally dissolved in chloroform.

#### Computational details

All calculations were performed using the Gaussian 03 program^51^. The hybrid functional B3LYP was employed at the DFT level of theory. Sulfur, nitrogen, carbon, oxygen and hydrogen atoms were described with a 6–31 G(d,p) double-z basis set. Phosphorus atoms were treated with LANL2DZ. Geometry optimizations were carried out under extremely tight criteria without any symmetry restrictions, and the nature of the extrema was verified with analytical frequency calculations. Thermal correction to Gibbs free energies was obtained at 298.2 K and 1.013 × 10^5^ Pa. The reference energy has been set to zero for the most stable ternary adduct of reactants.

#### Characterizations

NMR spectra were recorded on a Bruker (400 MHz) in CDCl_3_ with tetramethylsilane (TMS) as the internal reference. Size exclusion chromatography (SEC) was performed on Wyatt system equipped with a SSI 1500 pump and a Waters Styragel HR 2.5 μm, 300 mm × 7.8 mm column by using THF (0.7 mLmin^−1^) as eluent at room temperature. Matrix assisted laser desorption ionization time of flight mass spectra (MALDI TOF MS) were recorded at 25 kV on the Bruker mass spectrometer (ultraextreme). The polymer and the matrix 2,5-dihydroxybenzoic acid (DHB) were dissolved in CH_2_Cl_2_. 1 μl of the sample solution was piped onto the thin NaI crystal layer and dried in air. All mass spectra were collected by employing 500 individual laser shots. Transmission electronic microscopy (TEM) was conducted on a JEM-200cx operating at 200 kV. The sample was prepared by dipping the TEM copper grid to a dilute dispersion of silver nanoparticles in chloroform and solvent was evaporated at room temperature.

## Electronic supplementary material

supporting information
