# Supplementary material for: Organocatalyzed chemoselective ring-opening polymerizations
Source: Sci Rep. 2018 Feb 27;8:3734. doi: 10.1038/s41598-018-22171-6 (PMC5829214; doi:10.1038/s41598-018-22171-6)
Supplement: Supplementary file 1 — supporting information [file 41598_2018_22171_MOESM1_ESM.doc]

Supporting information

**Organocatalyzed chemoselective ring-opening polymerizations**

**Ning Zhu,^1,4^ Yihuan Liu,^1,4^ Junhua Liu,^5,6^ Jun Ling,^5^ Xin Hu,^2,4^ Weijun Huang,^1,4^ Weiyang Feng^1,4^ and Kai Guo*^,1,3,4^**

^1^College of Biotechnology and Pharmaceutical Engineering, ^2^College of Materials Science and Engineering, ^3^ State Key Laboratory of Materials-Oriented Chemical Engineering, ^4^ Jiangsu National Synergetic Innovation Centre for Advance Materials, Nanjing Tech University, Nanjing 211800, China

^5^ Department of Polymer Science and Engineering, Key Laboratory of Macromolecular Synthesis and Functionalization of the Ministry of Education, Zhejiang University, Hangzhou 310027, China

**^6^** Zhejiang Center for Drug & Cosmetic evaluation, Hangzhou 310012, China

a


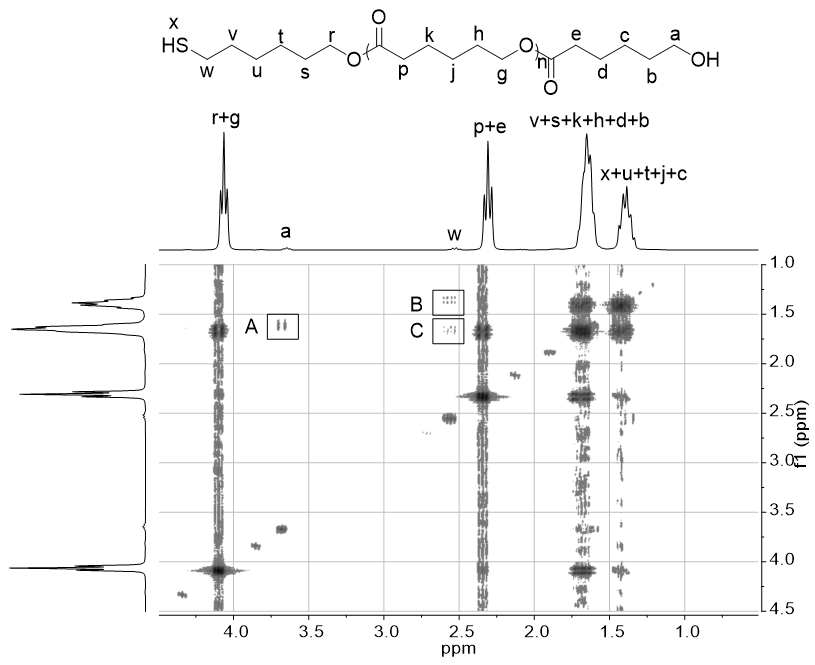

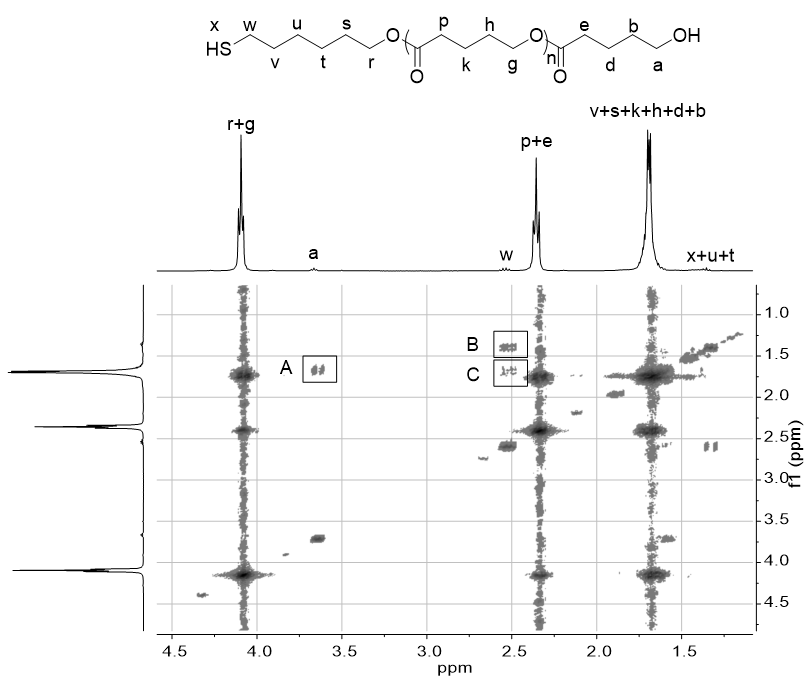


b

**Figure S1** ^1^H-^1^H COSY of PCLSH (Table 1, run 4) (a) and PVLSH (Table 2, run 10) (b).


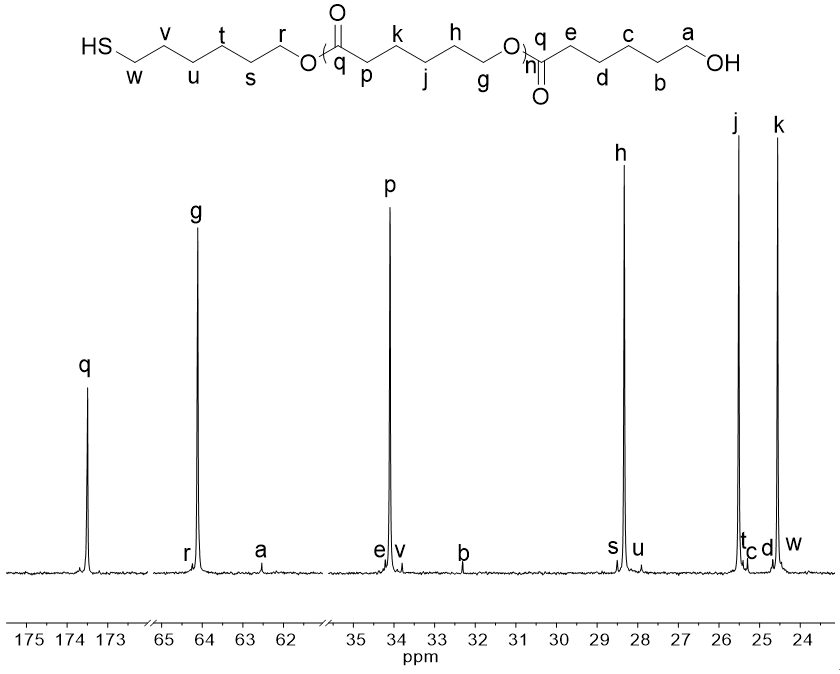


a

b


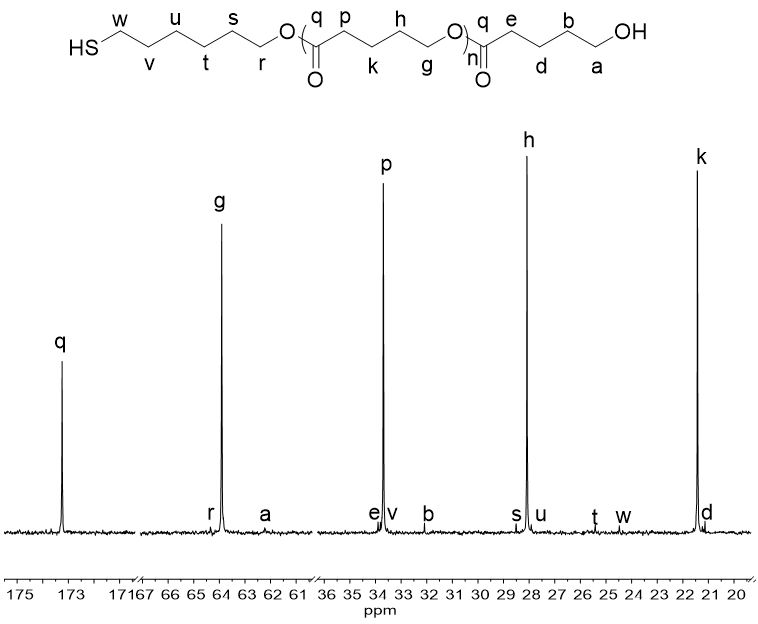


**Figure** **S2** ^13^C NMR of PCLSH (Table 1, run 4) (a) and PVLSH (Table 2, run 10) (b).


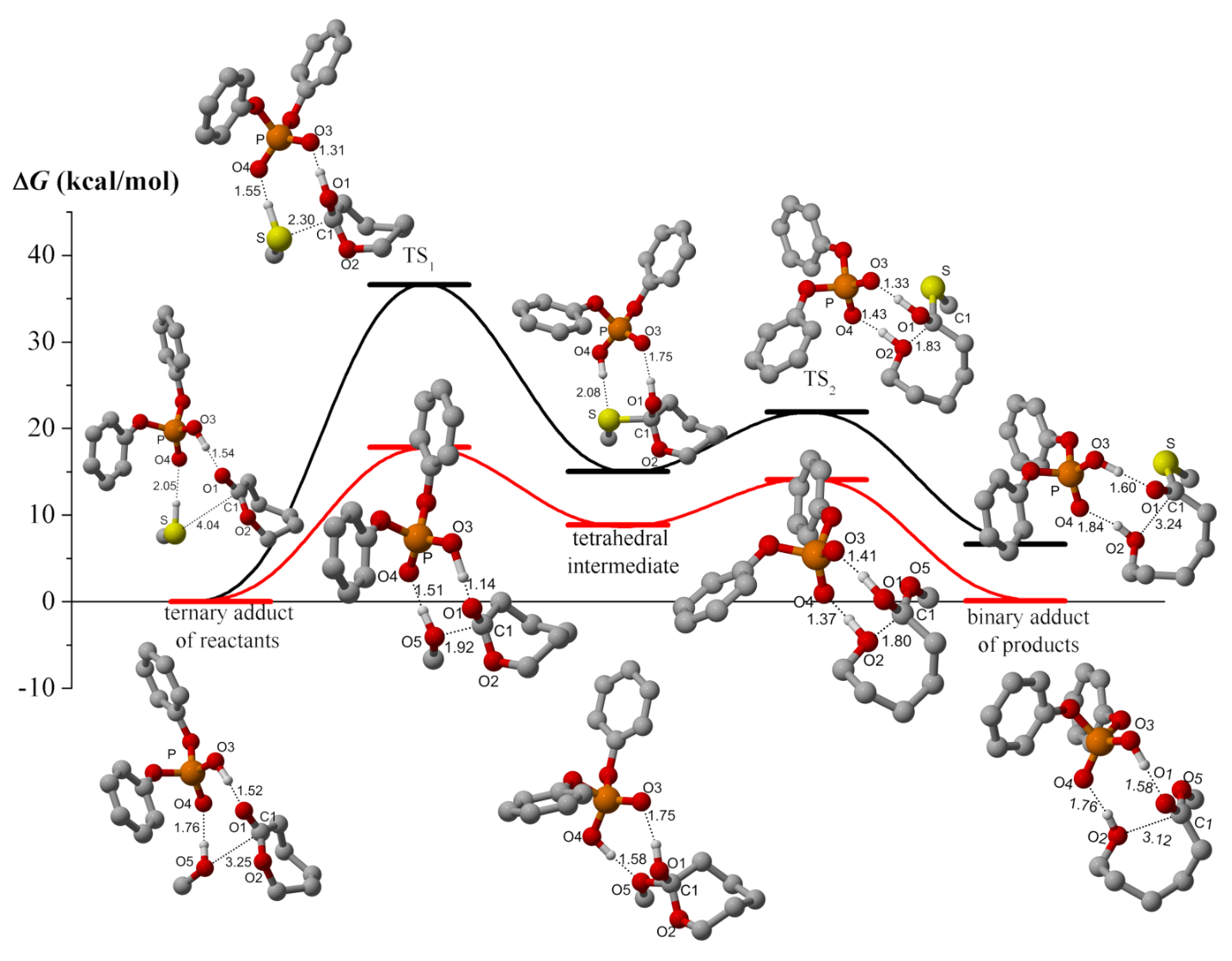


**Figure** **S3** Calculated Gibbs free energy profiles of the organocatalyzed ring opening reactions initiated by methanol (red line) and methanthiol (black line) respectively. All optimized geometries are illustrated by 3D models where some hydrogen atoms are neglected for clarity.

**Geometries information (reactions initiated by methanol)**

Ternary adduct of reactants

Gibbs free energy: -1271.337207H

C -2.08108400 0.01774100 2.73038600

C -3.02898900 1.00353500 2.99202600

C -4.31416200 0.93388300 2.46104200

C -4.65827400 -0.14839900 1.65585100

C -3.72836300 -1.15455900 1.39349100

C -2.44727000 -1.06707600 1.93398000

P -1.90548500 2.04751800 5.18485100

O -2.61060200 0.88215000 6.05779900

C -3.69033800 3.94824000 5.86819000

C -4.78378800 3.13348000 6.15220200

C -6.05479700 3.70567200 6.19061700

C -6.23051300 5.06947500 5.96323600

C -5.12168300 5.87023700 5.69082200

C -3.84688900 5.31265900 5.63871700

O -0.38802100 1.99347100 5.09290100

O -1.26963800 -1.27229600 5.97769700

C -0.07357600 -1.41019900 6.22625700

O 0.51066700 -2.46035100 5.66747100

C 1.92801700 -2.73273900 5.67170300

C 2.56326700 -2.73149600 7.05435100

C 3.02645800 -1.34440800 7.52184800

C 2.11991200 -0.21276000 7.03497500

C 0.62390100 -0.48378700 7.20311300

O 1.31229900 0.27946200 3.81476100

C 1.97990500 0.91430300 2.74538600

H 0.74579700 0.94417100 4.25599000

H -2.07092800 0.01340400 6.07544300

H 1.28272100 1.33225500 2.00516400

H 2.59463100 0.16298300 2.24006200

H 2.64334600 1.72386000 3.08354400

H 0.07479600 0.45774800 7.13786200

H 0.41197200 -0.89551100 8.20119700

H 2.31084100 0.01944500 5.98211000

H 2.35541600 0.70139400 7.59062400

H 4.04493700 -1.15546600 7.16188300

H 3.08414000 -1.33718700 8.61732900

H 3.41270200 -3.42435400 7.04453200

H 2.40548400 -2.02697700 4.98522000

H 1.83732800 -3.16371200 7.75427300

H 1.97666900 -3.72871500 5.22668000

H -4.63920000 2.07581300 6.34420800

H -6.91192600 3.07480900 6.40933900

H -7.22425300 5.50600000 5.99995100

H -5.24637300 6.93468100 5.51288000

H -2.96984400 5.91367300 5.42260500

H -5.02140500 1.72427900 2.68969400

H -5.65962800 -0.20676900 1.23845700

H -4.00144900 -2.00137800 0.77074200

H -1.71538200 -1.84457200 1.73557200

H -1.07191900 0.08646600 3.12728400

O -2.73359600 2.13950400 3.74069800

O -2.38112700 3.47854400 5.86284100

TS1

Gibbs free energy: -1271.308857H

C -2.56561900 0.09374700 1.95791000

C -3.48238100 0.97169400 2.53389400

C -4.84944000 0.83107900 2.30442100

C -5.30279700 -0.20443400 1.49201700

C -4.39968200 -1.10018200 0.91942900

C -3.03545000 -0.94661500 1.15806900

P -1.81481500 1.93720100 4.38321900

O -2.03724800 0.68112900 5.30237000

C -3.28570500 3.67551100 5.82976000

C -4.15131600 2.73215300 6.38117100

C -5.31635500 3.17355700 7.00734600

C -5.61091500 4.53281100 7.09501800

C -4.72852100 5.46261400 6.54524100

C -3.56571900 5.03858800 5.90830300

O -0.43465300 2.08022200 3.71379400

O -0.41921300 -1.08107700 5.06388100

C 0.77400000 -0.71293400 5.29964500

O 1.74307500 -1.65184100 5.17111300

C 1.64350100 -2.74606100 6.10491200

C 1.33997900 -2.28427600 7.52444700

C 2.23365400 -1.15246300 8.05037300

C 2.33191400 0.08742700 7.13524200

C 1.06410700 0.38347900 6.33029000

O 1.21162600 0.16396200 3.65314400

C 2.53653700 0.61509300 3.42112700

H 0.55737800 0.94900500 3.63227700

H -1.19617200 -0.25371400 5.19777100

H 2.60687600 1.02817900 2.40946100

H 3.19968500 -0.24737300 3.50556700

H 2.84400700 1.38659500 4.13874200

H 1.13708200 1.35534300 5.83530800

H 0.18436700 0.44798200 6.98037600

H 3.16406900 -0.03602800 6.43604000

H 2.56812600 0.96524100 7.74683800

H 3.24491200 -1.53283900 8.23926500

H 1.83666400 -0.85132000 9.02731100

H 1.42151200 -3.15661200 8.18414800

H 2.61942200 -3.23493100 6.04080800

H 0.28900600 -1.97487300 7.57381800

H 0.87561100 -3.44570900 5.75548300

H -3.90978500 1.67601500 6.32435500

H -5.99503600 2.44078400 7.43550300

H -6.51964000 4.86543500 7.58807900

H -4.94628000 6.52537200 6.60605700

H -2.86872800 5.74306500 5.46637300

H -5.53348000 1.53404900 2.76839800

H -6.36868600 -0.31360100 1.31170900

H -4.75683200 -1.91032400 0.29052200

H -2.32241300 -1.63567900 0.71421200

H -1.50128200 0.22554800 2.12335600

O -3.09762600 2.05188400 3.30964600

O -2.08333600 3.34644000 5.22281900

Tetrahedral intermediate

Gibbs free energy: -1271.323160H

C -3.77943800 0.90490500 0.96856200

C -3.70080800 0.76289100 2.35045700

C -4.07422300 -0.41983700 2.98312000

C -4.52351500 -1.48404000 2.20253500

C -4.61049700 -1.36080300 0.81694500

C -4.24034100 -0.16389700 0.20391700

P -2.03534000 1.80598800 4.14620300

O -2.14351500 0.74302400 5.24146000

C -3.10478300 3.92361200 5.38197200

C -3.75027500 3.23517500 6.40647500

C -4.77350700 3.87700500 7.10315900

C -5.13439800 5.18679700 6.79297700

C -4.46749200 5.86201600 5.77086200

C -3.45121700 5.23218500 5.05739300

O -0.71280700 1.75177500 3.22699500

O -0.15327100 -1.09271200 4.97250600

C 0.99766900 -0.48395900 5.39675400

O 2.07506500 -1.34463700 5.22092500

C 1.93929800 -2.67198500 5.73065500

C 1.37131500 -2.75777200 7.14213100

C 2.03325100 -1.83099000 8.16646800

C 2.07937000 -0.34887400 7.74199000

C 0.91067600 0.07758200 6.85039200

O 1.24106300 0.59586600 4.46715500

C 2.48611600 1.27550400 4.55081800

H 0.04170600 1.25076700 3.67617300

H -0.92437800 -0.51744700 5.17753900

H 2.47802500 2.02970000 3.76143100

H 3.31264400 0.57982600 4.38828600

H 2.61108500 1.77840800 5.51796000

H 0.85626300 1.16994100 6.79769400

H -0.04019700 -0.24766100 7.28506500

H 3.01489400 -0.14989200 7.20996100

H 2.09274000 0.28213300 8.63863000

H 3.05202800 -2.17557700 8.38384100

H 1.47562500 -1.92276300 9.10702400

H 1.45979100 -3.80047100 7.47285700

H 2.95841500 -3.07192900 5.69716900

H 0.29760300 -2.54692100 7.09534700

H 1.31672200 -3.26156800 5.04682200

H -3.45351000 2.21941200 6.64612900

H -5.28531600 3.34498500 7.90023500

H -5.92966700 5.67933000 7.34459900

H -4.74131100 6.88317300 5.52104400

H -2.92342400 5.73365300 4.25300200

H -4.00357000 -0.49759000 4.06235800

H -4.81165700 -2.41278200 2.68672800

H -4.96560700 -2.19343100 0.21689000

H -4.30477200 -0.06018300 -0.87548900

H -3.47866500 1.84339400 0.51509300

O -3.30272500 1.88624600 3.07770600

O -2.03943400 3.37845600 4.66755900

TS2

Gibbs free energy: -1271.314815H

C -3.23606200 -3.61667700 2.06815300

C -3.22283900 -2.55313100 2.96779400

C -3.64812400 -2.72288000 4.28583600

C -4.07389500 -3.98356200 4.70240400

C -4.09051100 -5.05760900 3.81352600

C -3.67491000 -4.86739900 2.49553500

O -2.84197200 -1.31413800 2.48185700

P -1.65842500 -0.43120300 3.28362600

O -2.17581000 0.14506600 4.62212600

O -1.56888900 0.84713600 2.21447800

C -1.27694200 0.71954100 0.86798400

C -1.78424700 1.72656300 0.04735900

C -1.50493600 1.70668500 -1.31593000

C -0.72815800 0.68520500 -1.86261600

C -0.22856900 -0.31421600 -1.02993200

C -0.49169200 -0.30482600 0.33939500

O -0.34783000 -1.27832700 3.31228400

O -0.66021800 0.12882700 6.57316500

C 0.58663000 0.16111300 6.22153600

O 0.92182500 -1.40886200 5.39809100

C 0.51549600 -2.63857500 6.02388900

C 0.73691200 -2.61605100 7.52498800

C 2.15477000 -2.24365300 7.96668700

C 2.66205500 -0.89309900 7.43109900

C 1.58197200 0.19236500 7.38119600

O 0.81047700 0.96913000 5.16428800

C 2.11963000 1.07611100 4.61118600

H -3.68795500 -5.69749000 1.79447200

H 2.04743100 1.18417500 7.35033400

H -1.30670400 0.19719500 5.73451000

H 0.97546000 0.17120800 8.29136000

H 3.47277900 -0.53904700 8.07837600

H 3.09387200 -1.02231000 6.43560200

H 2.85984900 -3.03112100 7.67270900

H 2.16531000 -2.22010100 9.06367800

H 0.48717800 -3.61371000 7.90627400

H 0.01230200 -1.93003600 7.97613700

H -0.53966600 -2.82828000 5.79697900

H 1.11067600 -3.43185000 5.55391800

H 2.84457900 1.42673300 5.35346900

H 2.44393500 0.11919700 4.19675400

H 2.03709000 1.81349400 3.81303600

H -2.90279900 -3.44618300 1.04971800

H -3.64908100 -1.87060500 4.95785500

H -4.40763700 -4.11980400 5.72767200

H -4.43086500 -6.03493100 4.14336000

H -0.09290600 -1.07216800 0.99443100

H -2.39283700 2.50674600 0.49277600

H -1.90229300 2.49200400 -1.95318900

H 0.38034700 -1.11338800 -1.44438700

H -0.51464200 0.66908700 -2.92738800

H 0.38150400 -1.32466500 4.47263200

Binary adduct of products

Gibbs free energy: -1271.337149H

C -3.43103300 -3.60991600 3.45433300

C -4.01921200 -2.38029800 3.16641500

C -5.40165700 -2.21657500 3.18766200

C -6.20997600 -3.30462800 3.50592100

C -5.64040900 -4.54167000 3.80731000

C -4.25454400 -4.68632400 3.78210900

O -3.27368500 -1.26988900 2.77856200

P -1.88277200 -0.82733300 3.57386000

O -2.40366100 -0.23969100 4.97793600

O -1.52541200 0.53973300 2.69851000

C -1.08515200 0.44676700 1.37883400

C -1.84729400 1.09367300 0.40999200

C -1.40743200 1.09574800 -0.91139400

C -0.22043300 0.45105100 -1.25864700

C 0.52935800 -0.19278200 -0.27557300

C 0.10763200 -0.19628500 1.05347600

O -0.78400600 -1.88569500 3.61363100

O -0.55743400 -0.34966400 6.80351900

C 0.49114700 0.23170500 6.54050000

O 1.82123400 -1.54902800 4.35416200

C 2.16877200 -2.80689400 4.90662500

C 1.79982700 -2.93199500 6.38563500

C 2.71891000 -2.16369200 7.34256700

C 2.88248600 -0.65487400 7.11828900

C 1.66395200 0.21691400 7.48998800

O 0.51488000 0.96039800 5.42696600

C 1.70851800 1.55041300 4.90350900

H -3.80281100 -5.64730500 4.01213300

H 1.99743600 1.25362600 7.62181800

H -1.68997800 -0.26987900 5.69796700

H 1.25569800 -0.11333800 8.44902700

H 3.70507400 -0.31076200 7.75714400

H 3.18707500 -0.46563500 6.08659400

H 3.72245400 -2.60716800 7.27807700

H 2.38439600 -2.33394500 8.37520000

H 1.84076600 -3.99365100 6.66537100

H 0.75541100 -2.62498400 6.51190900

H 1.68760100 -3.61663300 4.33990400

H 3.25328800 -2.92959900 4.77900300

H 2.27714600 2.07363500 5.67726200

H 2.31179000 0.77206800 4.43290000

H 1.36908700 2.26868400 4.15742500

H -2.35129200 -3.70998800 3.42292900

H -5.82097700 -1.24266000 2.95801900

H -7.28920500 -3.18114100 3.52340000

H -6.27345500 -5.38746400 4.05909100

H 0.68700700 -0.69279500 1.82411600

H -2.77057800 1.58099400 0.70489700

H -1.99849700 1.59923800 -1.67125800

H 1.45559600 -0.69592200 -0.53867600

H 0.11851500 0.45099200 -2.29053300

H 0.87087900 -1.59818000 4.12263400

**Geometries information (reactions initiated by methanthiol)**

Ternary adduct of reactants

Gibbs free energy: -1594.296161H

C -2.47080600 0.28505000 2.02381800

C -3.44941200 1.03393900 2.67182500

C -4.80310800 0.82687200 2.42371800

C -5.18201400 -0.15535000 1.51219700

C -4.21685900 -0.92591800 0.86351700

C -2.86588600 -0.70354400 1.12372800

P -2.05123600 1.81896100 4.79636100

O -2.67573300 0.58665800 5.64190700

C -3.61269400 3.70188900 5.94178600

C -4.66376300 2.89303100 6.36860000

C -5.88393600 3.48878400 6.68543000

C -6.04991700 4.86945000 6.59162400

C -4.98292700 5.66346100 6.17221600

C -3.76102500 5.08305300 5.84244700

O -0.57418300 1.71387300 4.45478800

O -1.12176700 -1.42522200 5.90508800

C 0.04195500 -1.47882000 6.29179500

O 0.62580700 -2.66758500 6.18065400

C 1.95912100 -3.01141900 6.61092900

C 2.32714300 -2.49589500 7.99374300

C 2.88847000 -1.06751000 7.98706000

C 2.23754000 -0.17930300 6.92636900

C 0.71263000 -0.25438800 6.88237900

C 2.79116500 1.23554400 2.33939700

H -2.02640100 -0.18971700 5.75566400

H 2.23641100 1.87315100 1.64875200

H 3.65680500 0.82251200 1.81776400

H 3.13937700 1.82709800 3.18816500

H 0.32727900 0.57919600 6.28568900

H 0.28526900 -0.13361100 7.88824300

H 2.63008900 -0.40952700 5.92909900

H 2.51198000 0.86494100 7.11150300

H 3.96972100 -1.09645200 7.80637100

H 2.75667500 -0.62468000 8.98178200

H 3.05809800 -3.18327600 8.43486000

H 2.66793600 -2.68366100 5.84304100

H 1.43341500 -2.56823000 8.62595400

H 1.93576600 -4.10318800 6.59936100

H -4.52487000 1.82067600 6.45285100

H -6.70792300 2.86233600 7.01592700

H -7.00330800 5.32385100 6.84463000

H -5.10020000 6.74080300 6.09537100

H -2.91879400 5.67924100 5.50720200

H -5.53410700 1.43356200 2.94783600

H -6.23716400 -0.32155000 1.31380200

H -4.51693100 -1.69459200 0.15741500

H -2.10622400 -1.29516200 0.62111900

H -1.42022000 0.47741300 2.21609000

O -3.12063700 2.06830800 3.54353700

O -2.34571000 3.20526300 5.65871900

S 1.77607400 -0.17795300 2.88507500

H 0.80304700 0.54500200 3.48561200

TS1

Gibbs free energy: -1594.255250H

C -2.92454900 0.30619700 1.25975500

C -3.71490700 0.86717000 2.26159600

C -5.06496300 0.54575000 2.37699700

C -5.62962900 -0.35487800 1.47732800

C -4.85037900 -0.93572500 0.47676700

C -3.50058000 -0.60307700 0.37416100

P -1.80259100 1.52183000 3.99205900

O -2.01926000 0.24529100 4.87261100

C -2.86002100 3.30917500 5.72516300

C -3.73688900 2.42183400 6.34875200

C -4.74419800 2.93283700 7.16629700

C -4.87271100 4.30512400 7.37207700

C -3.98184200 5.17782000 6.74749100

C -2.97627000 4.68480500 5.92100600

O -0.52759400 1.59522400 3.12396600

O -0.31170400 -1.46193400 5.02064300

C 0.84576100 -1.00279800 5.34527500

O 1.80047300 -1.93639500 5.60529000

C 1.51539300 -2.78690300 6.73741700

C 1.06510800 -2.00514300 7.96456900

C 1.92818300 -0.78484500 8.30977400

C 2.11552800 0.23186000 7.16044700

C 0.94517800 0.28363900 6.17713300

C 2.98932500 0.81654400 3.41786400

H -1.08817300 -0.67959100 4.94480100

H 3.09843800 1.33609100 2.46355900

H 3.91557900 0.28874000 3.64929500

H 2.77558100 1.54904600 4.19907000

H 1.01453800 1.16201900 5.52969400

H -0.01199000 0.39053800 6.70148600

H 3.02698100 -0.00340300 6.60261100

H 2.25876600 1.23161000 7.58506100

H 2.91435100 -1.11261500 8.66011500

H 1.45561400 -0.28230200 9.16206100

H 1.04838600 -2.69770400 8.81489700

H 2.46332500 -3.30166100 6.91535000

H 0.02456500 -1.69006100 7.82107600

H 0.76028800 -3.52694100 6.44982400

H -3.62434400 1.35363700 6.19628600

H -5.43115500 2.24364900 7.65024200

H -5.65971200 4.69164000 8.01299900

H -4.07131300 6.25028200 6.89748900

H -2.27830600 5.34569200 5.41739600

H -5.64903300 1.00528500 3.16753200

H -6.68303100 -0.60684200 1.56365400

H -5.29362800 -1.64102100 -0.22017900

H -2.88778000 -1.04610000 -0.40610500

H -1.88031000 0.59089300 1.18064100

O -3.21284600 1.81819300 3.13735900

O -1.80297700 2.90707800 4.92664200

S 1.65498600 -0.40665200 3.27867600

H 0.57903900 0.51303000 3.13768900

Tetrahedral intermediate

Gibbs free energy: -1594.272191H

C -3.85907500 1.47884000 0.90415400

C -3.65668800 0.98247800 2.18855700

C -3.80382900 -0.37192200 2.47666400

C -4.14665200 -1.24289200 1.44297900

C -4.35397900 -0.76495700 0.15048700

C -4.21294400 0.59736500 -0.11403800

P -2.09188700 1.70078100 4.22382400

O -2.17392100 0.44858000 5.09513900

C -3.25274300 3.55718800 5.75505100

C -3.97020500 2.68214300 6.56688900

C -5.03372100 3.18436700 7.31577800

C -5.36375700 4.53733500 7.26636500

C -4.62465200 5.39821600 6.45523800

C -3.56680900 4.91157600 5.69187100

O -0.73899400 1.88998300 3.35746600

O -0.33139100 -1.54677500 5.39836600

C 0.91261500 -0.95341600 5.38424000

O 1.89442600 -1.90087200 5.65122800

C 1.68389700 -2.74312100 6.79206400

C 1.24838900 -2.00882900 8.05338700

C 2.08656400 -0.77666400 8.40631300

C 2.22734400 0.24164600 7.25700000

C 1.01909800 0.29358800 6.31873100

C 2.83810500 0.30619400 3.60332200

H -0.11877900 1.09597600 3.41881000

H -1.02258400 -0.85051500 5.34619700

H 3.08960700 0.55669200 2.57005500

H 3.57960900 -0.39459500 3.99104300

H 2.83799700 1.22283400 4.19812400

H 1.05996000 1.20722900 5.71731500

H 0.08878200 0.35797600 6.89458400

H 3.11699300 0.00685800 6.66500800

H 2.38952600 1.24058400 7.67903600

H 3.08591400 -1.08309900 8.73990200

H 1.61560100 -0.28861600 9.26879600

H 1.27482500 -2.72662500 8.88322200

H 2.65549000 -3.22697700 6.93698500

H 0.19842400 -1.71903700 7.93901800

H 0.94849300 -3.51625000 6.54020300

H -3.69770500 1.63258900 6.60938900

H -5.60166100 2.50732200 7.94760300

H -6.19107000 4.91940700 7.85694800

H -4.87376600 6.45459600 6.40971800

H -2.98201400 5.55975200 5.04771100

H -3.64892500 -0.72853900 3.48910100

H -4.25696900 -2.30197300 1.65759900

H -4.62521300 -1.45007900 -0.64723100

H -4.37322300 0.97833400 -1.11862900

H -3.73591700 2.54123800 0.72215800

O -3.36611600 1.92176900 3.17973700

O -2.14634000 3.15381000 5.00835700

S 1.21157600 -0.49546600 3.57264500

TS2

Gibbs free energy: -1594.261249H

C -3.20936000 -3.59861000 2.15932600

C -3.19574000 -2.50907300 3.02721100

C -3.56957400 -2.65120800 4.36386500

C -3.94534700 -3.90996700 4.83156800

C -3.96253600 -5.00959500 3.97456400

C -3.59812800 -4.84741800 2.63773600

O -2.86764700 -1.27349100 2.49538800

P -1.65112400 -0.35673200 3.20384300

O -2.13106000 0.28717300 4.53605200

O -1.62722600 0.88258500 2.09333900

C -1.38956500 0.70284900 0.74061600

C -2.00571500 1.62313800 -0.10594300

C -1.78379300 1.54672100 -1.47787800

C -0.95709300 0.55460800 -2.00505500

C -0.34850000 -0.35781200 -1.14535300

C -0.55246500 -0.28972700 0.23233700

O -0.34153700 -1.19380800 3.23899900

O -0.73309500 0.09493700 6.51044300

C 0.53546200 0.18874500 6.28662900

O 0.99937900 -1.29412200 5.31523200

C 0.62726900 -2.61399300 5.78075700

C 0.61682800 -2.72492000 7.29266000

C 1.94244600 -2.37725700 7.97096300

C 2.49183000 -0.99263500 7.59798100

C 1.41578800 0.09689800 7.53808500

C 2.75699200 1.28006000 4.89221000

H -3.61208800 -5.69803800 1.96180500

H 1.88058900 1.08050700 7.66028800

H -1.36035300 0.21288600 5.61954100

H 0.71517300 -0.01567200 8.37278100

H 3.23659700 -0.69435000 8.34509000

H 3.01600800 -1.04092600 6.64042500

H 2.69748400 -3.13752000 7.73429800

H 1.79058100 -2.42472000 9.05674300

H 0.33963600 -3.75768500 7.53733700

H -0.18629300 -2.09328700 7.68627900

H -0.35714500 -2.86154800 5.37115100

H 1.36028100 -3.30474600 5.34479000

H 3.28726000 1.46223100 5.82994900

H 3.01000200 0.29661600 4.49415900

H 3.05455800 2.04438600 4.17058600

H -2.91585900 -3.44917100 1.12549500

H -3.57377800 -1.78012700 5.01123600

H -4.23957500 -4.02478000 5.87140300

H -4.26400200 -5.98552600 4.34392000

H -0.07057700 -0.98748100 0.90911700

H -2.65070500 2.38140400 0.32576000

H -2.26497500 2.26431700 -2.13678400

H 0.30078700 -1.13224700 -1.54511800

H -0.78850800 0.49437200 -3.07629400

H 0.47627800 -1.16873600 4.40930600

S 0.96280000 1.46017400 5.07225300

Binary adduct of products

Gibbs free energy: -1594.285557H

C -2.95018500 -3.62888700 3.50511700

C -3.68857100 -2.50364800 3.14567700

C -5.07806600 -2.49131500 3.23281200

C -5.73967900 -3.62717100 3.69170900

C -5.01801600 -4.76054100 4.06646400

C -3.62756500 -4.75424300 3.97327400

O -3.09248200 -1.36076900 2.61872000

P -1.75422300 -0.66896400 3.32438300

O -2.31031500 -0.01331100 4.68792300

O -1.62298800 0.65934600 2.34141400

C -1.35125900 0.56400500 0.98050300

C -1.94966600 1.53103800 0.17649700

C -1.68758000 1.53647000 -1.19066100

C -0.84041200 0.58017300 -1.75045800

C -0.25033100 -0.37949100 -0.93043500

C -0.49335500 -0.39285300 0.44254400

O -0.52733400 -1.56952900 3.42517500

O -0.61700000 -0.04547700 6.66964400

C 0.51620800 0.42746600 6.60146000

O 2.03805600 -1.56476700 4.54184400

C 2.00784400 -2.86507100 5.10234100

C 1.28633700 -2.92780400 6.44978400

C 2.08078400 -2.35096900 7.62728100

C 2.57721500 -0.90402300 7.51084800

C 1.51799800 0.20105800 7.70959000

C 2.65816100 1.66829900 5.16599600

H -3.05830600 -5.63434200 4.25929800

H 2.03193200 1.14955100 7.90130800

H -1.64723100 -0.02553500 5.44635000

H 0.91332700 -0.02987300 8.59370700

H 3.32224300 -0.74126600 8.29960400

H 3.09109600 -0.76553300 6.55740200

H 2.96917500 -2.98137200 7.77361000

H 1.48923300 -2.45344300 8.54749500

H 1.06871400 -3.98028500 6.67834000

H 0.31475700 -2.43259100 6.34909100

H 1.53591100 -3.57034700 4.40318700

H 3.05162700 -3.18705100 5.22178400

H 3.08912200 1.99762200 6.11347600

H 3.08578200 0.71868600 4.84111300

H 2.84923000 2.43461100 4.41144600

H -1.86906500 -3.61208900 3.41923300

H -5.61672200 -1.59539600 2.94268800

H -6.82383600 -3.62228900 3.76085600

H -5.53713600 -5.64351500 4.42748800

H -0.02638800 -1.12853200 1.08898000

H -2.61094900 2.25969100 0.63360300

H -2.15342500 2.28948600 -1.82016200

H 0.41415700 -1.12617700 -1.35622300

H -0.64126100 0.58411800 -2.81801600

H 1.16622900 -1.42001100 4.12989500

S 0.85324100 1.51421700 5.24604800


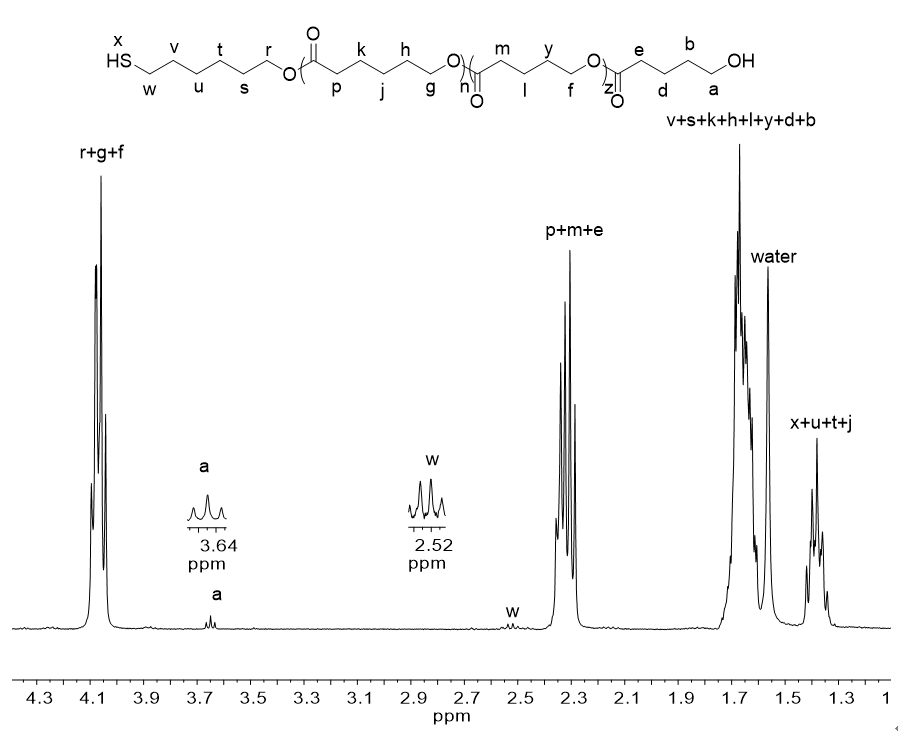


a


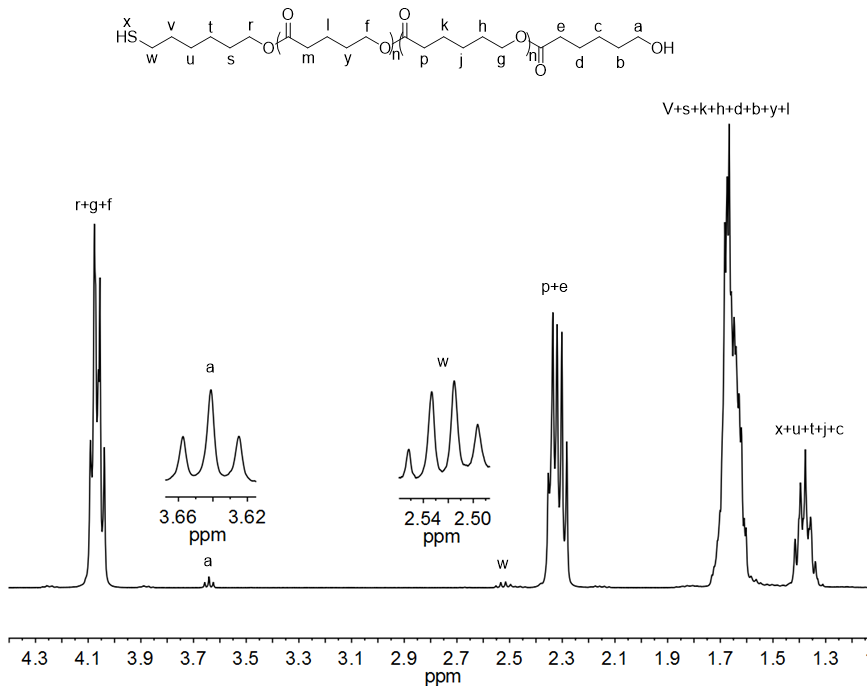


b

**Figure** **S4** ^1^H NMR of PCL-*b*-PVLSH (a) and PVL-*b*-PCLSH (b).


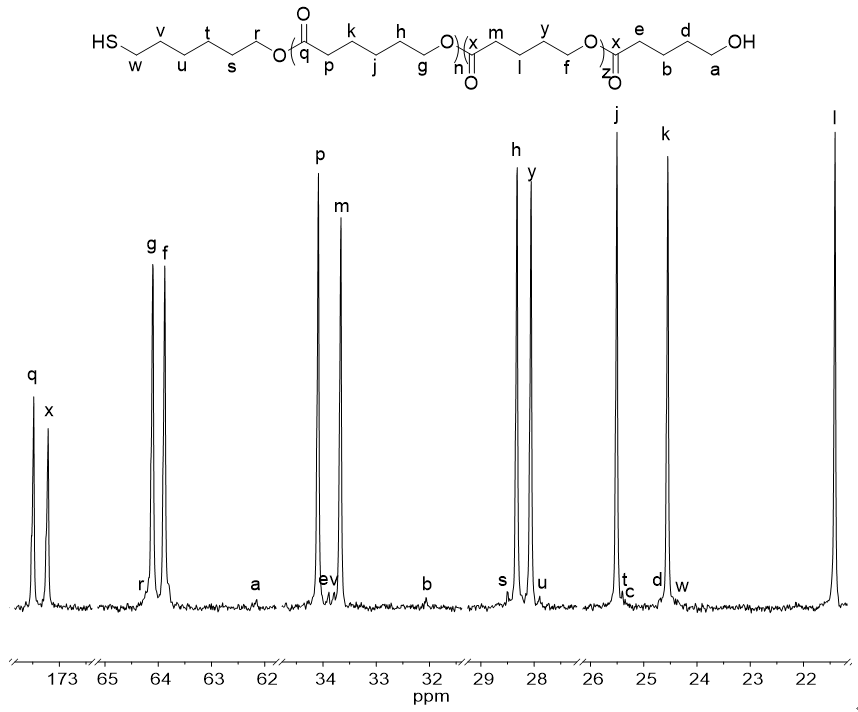


a


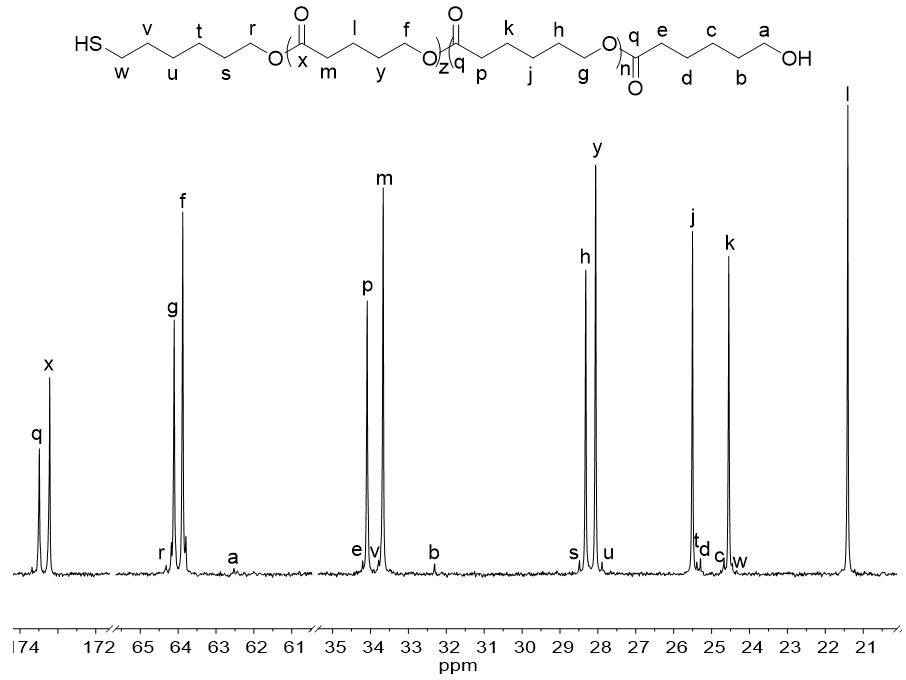


b

**Figure** **S5** ^13^C NMR of PCL-*b*-PVLSH (a) and PVL-*b*-PCLSH (b).
